# Health-related quality of life and the predictive role of sense of coherence, spirituality and religious coping in a sample of Iranian women with breast cancer: a prospective study with comparative design

**DOI:** 10.1186/s12955-015-0229-1

**Published:** 2015-03-28

**Authors:** Camelia Rohani, Heidar-Ali Abedi, Ramesh Omranipour, Ann Langius-Eklöf

**Affiliations:** Department of Health, Shahid Beheshti University of Medical Sciences School of Nursing and Midwifery, Valiasr st., Niayesh Crossroad, Tehran, 1985717443 Iran; Department of Nursing, Islamic Azad University Nursing and Midwifery School, Isfahan (Khorasgan) Branch, Isfahan, 8153653791 Iran; Department of Surgical Oncology, Cancer Institute, Tehran University of Medical Sciences, Tehran, 1419733141 Iran; Department of Neurobiology, Care Sciences and Society, Karolinska Institutet, Alfred Nobels Alle’ 23, Huddinge, 14183 Sweden

**Keywords:** Health-related quality of life, Breast cancer, Sense of coherence, Spirituality, Religious coping

## Abstract

**Background:**

There is disagreement among studies of health-related quality of life (HRQoL) changes in breast cancer patients over time. Reportedly, assessment of HRQoL prior to diagnosis may be crucial to provide a clear point of comparison for later measurements. The aims of this study were (1) to investigate changes in HRQoL, sense of coherence (SOC), spirituality and religious coping in a group of women with breast cancer from the pre-diagnosis phase to 6 months later in comparison with a control group, and (2) to explore the predictor role of SOC, spirituality, and religious coping within the breast cancer group at the 6-month follow-up.

**Methods:**

A sample of women with breast cancer (n = 162) and a matched control group (n = 210) responded to the following instruments on both occasions: the European Organization for Research and Treatment of Cancer QLQ-C30, the SOC Scale, the Spiritual Perspective Scale and the Brief Religious Coping Scale. A series of General Linear Model (GLM) Repeated Measures was used to determine changes between the groups over time. Also, Multiple Linear Regression analyses were applied to each of the HRQoL dimensions, as dependent variable at the 6 months follow-up.

**Results:**

Physical and role function, fatigue, and financial difficulties were rated worse by the women with breast cancer during the first 6 months in comparison to the controls, which was both a statistically (p < 0.001) and clinically significant difference. Women had better scores for global quality of life (p < 0.001), and emotional functioning (p < 0.01) during the same period of time. The degree of SOC (p < 0.01) and baseline ratings of several dimensions of HRQoL (p < 0.05) were the most important predictors of HRQoL changes.

**Conclusions:**

Collecting HRQoL data before a final diagnosis of breast cancer is important to identify women at risk of deterioration in HRQoL during and after treatment. Special attention should be paid to physical and role functioning impairment, fatigue, and financial difficulties experienced by these women. These results underscore that the degree of SOC may be more important as a predictor for HRQoL changes in this sample than spirituality and religious coping.

## Background

Breast cancer is the most common cancer in women, both in high-income and low/middle- income countries [1]. It is a prevalent cancer in Iranian women, ranking first among malignancies and fifth among causes of death [2]. It seems that in Iran, like in other middle-income countries, breast cancer appears in women at least one decade younger than their counterparts in high-income countries [3]. At the national level in Iran, mammography screening for breast cancer is not routinely scheduled [4], so early detection of breast cancer remains an essential challenge [5]. Being diagnosed with breast cancer may be experienced as a psychosocial transition connected with negative as well as positive consequences [6]. Thus, living with breast cancer often involves changes in life, especially regarding Health-related Quality of life (HRQoL) [7,8].

Commonly HRQoL is defined as multidimensional, contextual, dynamic, and subjective concept which is related to a medical condition [9]. It is an important outcome variable in cancer patients [10], because it affects the prognosis, and it can be used for patient monitoring, clinical decision-making, treatment, symptom management and planning of supportive care interventions [11]. It is suggested that when measuring HRQoL in patients with cancer a complex set of physical, mental and social dimensions and disease-specific symptoms, should be evaluated [12].

HRQoL changes in breast cancer patients over time show discrepant results [13-15]. Some studies show that follow-ups within 6 months after diagnosis yield significant improvements in most HRQoL dimensions over time [16,17], although some problems may still remain (e.g., poor body image and an uncertain view of the future, in addition to systemic therapy side effects) [18]. Other studies show reduced [15,19] or stable HRQoL [13] within the first 6 months after treatment. Most prospective HRQoL studies have focused on a baseline at the time of the cancer diagnosis [13,20] or the post-diagnosis period [21,22].

Understanding the ways patients cope with a cancer diagnosis and treatment is important in determining their HRQoL [9]. There are several ways to measure coping with life strain. The concept of sense of coherence (SOC), although being an area under discussion, has a broad theoretical base and a growing body of empirical evidence, supporting its utility as a determinant for successful coping [23]. The concept of SOC is defined as an individual’s global view of life, and as an inner resource for coping with stressful life events, and thus does not refer to a particular coping strategy [24]. The SOC is built upon a dynamic interrelation between three components: comprehensibility, manageability, and meaningfulness. Individuals with a higher SOC try to perceive their life as more manageable, meaningful, and comprehensible, and these abilities are hypothesized to decrease the tensions in life [24]. The theory behind the SOC suggests that in adulthood (to about 30 years of age) it is a stable trait and may only fluctuate when radical life events occur [24]. The concept is operationalized into a self-administered instrument, the SOC scale which measures an individual’s SOC. Both the validity and reliability, including cross-cultural adaptation of the scale (translated to 33 languages) have been supported in numerous studies [25], including cancer populations [26-28]. Some studies suggest that the SOC changes over time in adults [29,30]. Other studies, however, support clearly the stability of the SOC [31,32] and a review concludes that variations over time are small in adults [25]. Studies show that a high SOC serves as a determinant for successful adaptation to stressful situations, and is correlated to better health and quality of life within different samples [33-35], also in women with breast cancer [26].

Other personal inner resources that are also suggested to be important when determining HRQoL are spirituality [36,37] and religious coping [38,39]. Although the importance of spirituality and religious coping may be different across various cultures [40], there is growing evidence that these concepts may be main resources when an individual is confronted with a potentially life-threatening disease [41-43]. Spirituality has been explained as a construct that expands further than religiousness. Through spirituality individuals attempt to perceive their world, themselves, their requirements and their connection to self, others, nature and God [44]. Reed [45] underlines that spirituality is a form of self-transcendence, and defines it as an individualized awareness of one’s inner self and a sense of conjunction with a powerful dimension or purpose. She developed the spiritual perspective scale (SPS) for measuring one aspect of spirituality, spiritual perspective, in a way that could be meaningful in different settings and health conditions [46]. The SPS refers mainly to spiritual behaviors and beliefs, and does not include subscales which refer to well-being [47].

Religious coping, a close but not interchangeable concept, was defined as efforts to perceive and manage life stressors in a way linked to the notion of God or divinity [38]. Religious coping consists of a positive dimension, reflecting strategies to maintain a secure relationship with God/a higher power, and a negative dimension, reflecting a religious struggle [48]. Pargament [48] developed the religious coping (RCOPE) scale for measuring of this concept from his theory. The RCOPE scale represents a different approach to religious assessment. This scale is not measuring intrinsic and extrinsic religious orientation, unlike earlier assessments of religiosity, even though it may be correlated to these [38]. Generally, positive religious coping was associated with improvements in health, and negative religious coping predictive of declines in health [49]. Patients who struggle religiously over time may be at risk for health-related problems [49]. Negative religious coping has been recognized as a robust predictor of health-related outcomes [38]. Spirituality and religious coping correlate to physical and mental health [38,50] and have been found to predict HRQOL among cancer survivors [51,52].

The differences between studies on how women with breast cancer perceive HRQoL may depend on how the term HRQoL is defined and measured [9,53]. Measurement of HRQoL prior to diagnosis may be crucial to provide a clear point of comparison for later measurements, and could make a more reliable interpretation of the results possible [54]. Furthermore, to cover all dimensions of HRQoL related to theory and the context of culture, the use of several instruments is required [9]. In numerous studies in Iran the importance of spiritual and religious beliefs among patients with breast cancer has raised interest [55,56], but no previous studies have applied the concept of SOC in this population to date. Therefore, the main aims of this study were to investigate changes in HRQoL, SOC, spirituality and religious coping in a group of women with breast cancer from the pre-diagnosis phase to 6 months later, compared with a matched control group and to explore the predictor role of SOC, spirituality, and religious coping within the breast cancer group at the 6-month follow-up. HRQoL was measured by the European Organization for Research and Treatment of Cancer (EORTC) QLQ-C30, a specific questionnaire for cancer patients [9], and the SOC, spirituality and religious coping were measured by standardized scales. Additionally, we discuss the predictors of HRQoL dimensions at the 6-month follow-up with focus on baseline HRQoL data and the SOC, spirituality and religious coping.

## Methods

### Design

This study was longitudinal with a prospective and comparative design at two time points: baseline (pre-diagnosis phase of breast cancer) (T1) and 6 months post pre-diagnosis (T2).

### Samples

Inclusion criteria for both groups consisted of having sufficient knowledge of the Persian language to answer the questionnaires and no previous cancer history.

#### Breast cancer group

**Figure 1:**
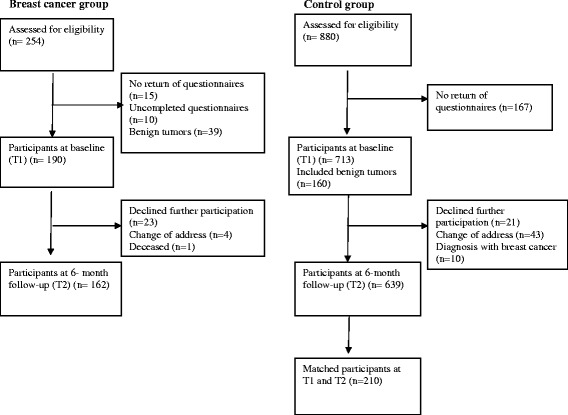
**Flow chart of sampling for the breast cancer and control groups.**

During several months, five days a week after checking the admission books in the nursing stations, a prospective sample of 254 women with an operable lump in the breast or other symptoms indicative of breast cancer were approached by the first author before surgery (the pre-diagnosis phase) at the surgical wards of two educational hospitals in Tehran. One of the hospitals is a center for cancer patients, with 1300 beds and 4300 employees, the other is a center for women’s diseases, with 111 beds and 344 employees. Both hospitals together treat around 750 breast cancer patients annually, including newly diagnosed and follow-up patients. In total 254 women met the inclusion criteria, but 92 participants (36%) dropped out by the end of the 6-month follow-up, leaving a final sample of 162 patients (64%) (Figure 1). At baseline, both the patients and the authors were blind to the final diagnosis. The breast cancer diagnosis was confirmed with a quick pathology report during surgery. This report was thereafter controlled in detail, and the final result was given to the patients two to three weeks later. Women with a confirmed diagnosis of breast cancer were included in the study’s follow-up, and those with benign results were excluded (n = 39). There was no exclusion from the study with regard to type of breast cancer, disease stage, and having or not having a pre-surgery biopsy report. After surgery, the patients underwent a single or combined treatment modality (chemotherapy, radiotherapy and hormonal therapy), based on National Comprehensive Cancer Network Guidelines [57].

#### Control group

During several months, using consecutive odd queue numbers, three days of a week, in total 880 women were randomly selected by a trained colleague from the Mammography Centers at the same hospitals before undergoing mammography/breast ultrasonography. These women visited the centers by referral from a physician, based on their own or the physician’s initiative. Of the 880 eligible women, 167 participants (19%) did not return the questionnaires, 21 (2%) declined further participation, 43 (5%) had changed their address and 10 (1%) were diagnosed with breast cancer, leaving 639 women (73%) at the 6-month follow-up. A sub-sample of 210 participants was drawn by frequency matching, (see Statistical analyses) (Figure 1).

### Measurements

The European Organization for Research and Treatment of Cancer QLQ-C30 (EORTC QLQ-C30) version 3 (30 items) is a core cancer-specific questionnaire, and includes a global health status/quality of life scale, five functional scales (physical, role, emotional, cognitive, and social functioning), three symptom scales (fatigue, pain, and nausea/vomiting) and six single items (appetite loss, insomnia, dyspnea, constipation, diarrhea, and financial difficulties). All of the scales and single-item measures are transformed to scores ranging from 0 to 100. A high score represents a high global quality of life and functioning or a high level of symptoms. The validity and reliability of the instrument has been supported in different countries, including Iran [58,59]. The ability of the EORTC QLQ-C30 to detect HRQoL differences in the normal population has also been confirmed [60]. In our study, all Cronbach’s alpha coefficients for the EORTC QLQ-C30 scales at T1 were on the suggested level (>0.60) [9] in both groups, except for three scales in the breast cancer group: role functioning (0.46), cognitive functioning (0.44) and nausea/vomiting (0.44). The low coefficients for these scales could be due to the low number of items on these scales [61], and this is consistent with some previous findings as well [13,59]. However, all scales of the EORTC QLQ-C30 in the breast cancer group met the criterion (Cronbach’s alpha coefficient > 0.60) at T2.

The Sense of Coherence (SOC) scale includes 13 items for which the respondents indicate agreement or disagreement on a seven-point scale. The total score can range from 13 to 91, with higher scores representing a higher SOC [24]. The SOC scale has demonstrated satisfactory psychometric properties with cross-cultural applicability [25], including validity of the Iranian version [62]. In the present study Cronbach’s alpha coefficients were > 0.77 in both groups on both occasions.

The Spiritual Perspective Scale (SPS) is a 10-item scale that measures the extent to which individuals hold certain spiritual beliefs and engage in spiritually related behaviors [46]. Each item ranges from 1 to 6, and the items are summed to one scale summary (10–60 points), where a higher score represents a stronger spiritual perspective. The scale is organized to reflect spiritual behaviors (4 items) and spiritual beliefs (6 items) [63]. One example of the items of spiritual behaviors is: “How often do you engage in private prayer or meditation?.” Responses are: 1 (not at all), 2 (less than once a year), 3(about once a year), 4 (about once a month), 5 (about once a week), and 6 (about once a day). One example of the spiritual belief items is: “My spiritual views have had an influence upon my life.” Responses are: 1 (strongly disagree), 2 (disagree), 3 (disagree more than agree), 4(agree more than disagree), 5 (agree), and 6 (strongly agree). None of the items refers to a specific religion [63]. The psychometric properties of this instrument have been demonstrated within different samples [64], including the Iranian version [62]. In the present study Cronbach’s alpha coefficients were > 0.78 in both groups on both occasions.

The Brief Religious Coping (Brief RCOPE) scale is used to assess religious coping. It comprises 14 items, each on a four-point rating scale from “not at all” to “a great deal”, that distinguish between positive and negative religious styles. Seven items reflect positive religious coping, including strategies such as looking for spiritual support and benevolent religious reappraisals, e.g. “Looked for a stronger connection with God”. Seven items reflect negative religious coping and contains items related to spiritual struggle, such as “Wondered whether God had abandoned me” [48]. The scoring range for each scale is from 7 to 28: the higher the score, the stronger the positive/negative religious coping, respectively. The validity and reliability of the Brief RCOPE has been reported [48], including an Iranian version of the scale [62]. Cronbach’s alpha coefficients of both scales were 0.80 and above in both samples on both occasions.

Demographic data for both groups were collected at the first visit by questions about age, marital status, working status, and educational level. Clinical data for the breast cancer group was obtained from medical records (menopause, comorbidity, cancerous body side, biopsy before surgery, type of surgery, stage, and treatment type), and for the controls by a self-reported questionnaire (menopause and comorbidity).

### Data collection

The study was approved by the National Ethical Board of Research at the Ministry of Health and Medical Education of Iran (P/391-31). The participants in both groups were given verbal and written information about the study, and written informed consent was obtained from all of them. The information explained the study objectives of comparing women diagnosed with breast cancer, with those without breast cancer. All women were in a more or less vulnerable situation with a potential breast cancer diagnosis. The researcher emphasized voluntariness and the right to withdraw from further participation at any stage of the study.

Baseline data for the breast cancer group were collected by the EORTC QLQ-C30, the SOC scale, the SPS, and the Brief RCOPE scale on days 1–14 before surgery. In the controls, the same assessments were completed before undergoing mammography/breast ultrasonography examination at the Mammography Centers. At baseline, the questionnaires were collected at the hospital. Six months later, the same questionnaires accompanied by an information letter and a pre-stamped envelope were sent via post to the participants in both groups.

### Statistical analyses

A priori power analysis by G^*^ Power 3 version 3.1.5.1, with an estimated effect size of 0.3 according to earlier studies [22,65], showed that a minimum of 235 subjects should be included in the study for each group to detect changes by a power of 90% at 0.05 significant level. Estimating about 10% drop-out rate, 254 women suspected of having breast cancer were approached. During the same time, women in the control group were randomly recruited, and the final sample included 880 women, in order to facilitate post stratification and matching with the breast cancer group. A posteriori power analysis showed that the remaining samples by the end of the study detected changes by a power of 80% at 0.05 significant level in the breast cancer (n = 162) and control groups (n = 210).

According to the initial analysis of the data, differences between the two groups appeared, and therefore, after post stratification, frequency matching was applied [66]. The sample size allowed for six matching variables: age, education, marital and working status, menopausal status, and existence of comorbidities. Outcome variables met the normality assumptions by P-P plots. For all analyses, the significance level was set at p < 0.05. Mean differences within the groups were estimated by the dependent Student’s t-test from T1 to T2, and between the groups by the independent Student’s t-test.

All scales were linearly transformed to 0–100 scores, and the interaction effect between time (T1 vs. T2) and group (breast cancer group vs. the controls) on the outcome variables was estimated. Therefore, a series of general linear model (GLM) repeated measures were run with the number of comorbidities as a covariate (still significantly different between the groups after matching, see results section), to determine adjusted mean score differences between the groups over time. The degree of clinical changes and their directions was calculated from the results of the GLM analyses by adjusted mean differences between the groups. Clinical changes were interpreted in terms of small (5–10), moderate (11–19), or large (≥20) [67].

Ahead of multiple linear regression (MLR) analyses, a correlation matrix was generated between demographic and clinical variables and HRQOL variables in the breast cancer group at T2 to explore which of them should be included as independent variables. Those that resulted with statistical significance were age, education, surgery type, cancer stage, and treatment modalities. The SOC scale, SPS and the Brief RCOPE scale scores and baseline scores of HRQoL dimensions were also independent variables, based on their earlier confirmed predictor value in HRQOL studies. Subsequently, independent variables were entered into the MLR analyses in four blocks: demographic variables (dichotomized, except for age), clinical variables (dichotomized), the SOC, the SPS, and the Brief RCOPE scale scores at T2, and baseline score of HRQoL dimensions. MLR analyses were run with each of the HRQoL dimensions at T2 as dependent variables. Multicollinearity among the SOC, SPS and Brief RCOPE scores, in addition to the rest of MLR assumptions (normal distribution of the residuals and homoscedasticity) were tested and fulfilled [68]. Statistical analyses were conducted using the SPSS version 17.0 (SPSS Inc., Chicago, IL, USA, 2008).

## Results

### Descriptive data

Demographic and clinical characteristics of the two groups are summarized in Tables 1 and 2. The mean age of the breast cancer and control group were, respectively M = 46.1, SD = 9.8 and M = 46.6, SD = 8.4. Most of the patients and the controls had an education at less than university level (68.5%, 66.2%), were married (79.6%, 81%), housewives (59.9%, 55.7%), and in a premenopausal stage (63%, 67.6%) (Table 1).

**Table 1:** **Demographic and clinical data in the breast cancer (n = 162) and control groups (n = 210)**

**Demographic & clinical variables**	**Breast cancer group n (%)**	**Control group n (%)**	**df**	**p value**
**Age in years, Mean (SD)**	46.1 (9.8)	46.6 (8.4)	370	.071^a^
**Marital status**			2	.572^b^
Single	11 (6.8)	24 (11.4)		
Married	129 (79.6)	170 (81.0)		
Divorced/Widowed	22 (13.6)	16 (7.6)		
**Education**			1	.557^b^
Primary school	23 (14.2)	35 (16.7)		
Secondary school	27 (16.7)	23 (11.0)		
High school	4 (2.5)	6 (2.9)		
Diploma	57 (35.2)	75 (35.7)		
University	51 (31.5)	71 (33.8)		
**Working status**			2	.356^b^
Housewife	97 (59.9)	117 (55.7)		
Employed	51 (31.5)	65 (31.0)		
Retired	14 (8.6)	28 (13.3)		
**Menopause at beginning of the study**			1	.349^b^
Yes	60 (37.0)	68 (32.4)		
No	102 (63.0)	142 (67.6)		
**Comorbidity** ^c^			1	.935^b^
Yes	78 (48.1)	102 (48.6)		
No	84 (51.9)	108 (51.4)		
**Number of comorbidities**			1	.001^b^
One	42 (26.0)	30 (14.3)		
More than one	36 (22.0)	72 (34.3)		

^a^Differences between groups’ proportions were tested by the Independent Student’s t-test.

^b^Differences between groups’ proportions were tested by the Chi-Square.

^c^Comorbidity: long-standing diseases such as diabetes, hypertension, and musculoskeletal problems.

Furthermore, most patients underwent a mastectomy (55.6%) and most had a combined treatment modality (84%) after surgery (Table 2). At the 6-month follow-up, 33% of the patients were in the treatment end point phase and the rest of them have been free of treatments for at least one week (Table 2).

**Table 2:** **Specific clinical characteristics of the breast cancer patients (n = 162)**

**Variable**	**n**	**Percent (%)**
**Breast cancer**		
One side	159	98.1
Two sides	3	1.9
**Biopsy before surgery**		
Yes	96	59.3
No	66	40.7
**Type of surgery**		
Breast conservation	72	44.4
Mastectomy	90	55.6
**Stage**		
0	4	2.5
I	37	22.8
II	80	49.4
III	39	24.1
IV	2	1.2
**Chemotherapy**		
Yes	128	79.0
No	34	21.0
**Radiotherapy**		
Yes	122	75.3
No	40	24.7
**Hormonal therapy**		
Yes	111	68.5
No	51	31.5
**Combined & single treatment modality**		
CT + RT + HT	62	38.3
CT + RT	35	21.6
CT + HT	16	9.9
RT + HT	23	14.2
CT	15	9.3
RT	1	0.6
HT	10	6.2
**Treatment at the 6-month follow-up** ^**a**^		
Ongoing treatment	53	32.7
Treatment ended 1–2 weeks before follow-up	75	46.3
Treatment ended more than 1 month before follow-up	34	21.0

CT: Chemotherapy; HT: Hormonal therapy; RT: Radiotherapy.

^a^It includes just CT and RT.

The groups differed in several scale scores at T1 (Table 3). The patients reported significantly lower global quality of life, emotional and social functioning, and more appetite loss and financial problems than the controls, combined with higher physical, role, and cognitive functioning, and lower constipation. The breast cancer group also had higher scores than the controls on the SOC, spirituality, and positive religious coping at T1.

**Table 3:** **The results of the dependent and independent Student’s t-test in the breast cancer (n = 162) and control groups (n = 210) at baseline (T1) and at the 6-month follow-up (T 2)**

**Variables**	**Range**	**BC** ^**1**^ **group mean (SD)**		**Differences within BC group** ^**3**^	**C** ^**2**^ **group mean (SD)**		**Differences within C group** ^**3**^	**Differences between groups** ^**4**^ **at T1**	**Differences between groups** ^**4**^ **at T2**
		**T1**	**T2**	**(p value)**	**T1**	**T2**	**(p value)**	**(p value)**	**(p value)**
**EORTCQLQ-C30:**									
Global quality of life	0-100	58.1(20.1)	68.7 (18.5)	**.000**	70.1 (21.6)	72.4 (18.0)	.098	**.000**	.053
**Functional scales:**									
Physical	0-100	93.8 (9.6)	77.4 (16.3)	**.000**	85.5 (12.6)	85.2 (12.7)	.713	**.000**	**.000**
Role	0-100	94.6 (12.3)	81.9 (19.3)	**.000**	90.0 (16.4)	88.2 (16.8)	.160	**.002**	**.001**
Emotional	0-100	55.2 (27.4)	65.3 (23.8)	**.000**	72.5 (22.8)	74.0 (21.6)	.335	**.000**	**.000**
Cognitive	0-100	87.9 (15.1)	83.3 (18.2)	**.009**	82.5 (20.6)	84.0 (19.2)	.261	**.004**	**.716**
Social	0-100	82.2 (21.3)	74.8 (24.6)	**.001**	93.7 (14.5)	93.3 (15.0)	.745	**.000**	**.000**
**Symptom scales:**									
Fatigue	0-100	18.3 (18.4)	31.6 (23.3)	**.000**	21.0 (16.4)	22.5 (19.3)	.234	.130	**.000**
Nausea/vomiting	0-100	4.2 (9.3)	7.4 (16.2)	**.024**	3.5 (9.1)	3.3 (9.7)	.840	.452	**.005**
Pain	0-100	15.1 (17.4)	25.0 (21.9)	**.000**	15.9 (19.9)	17.2 (19.9)	.342	.699	**.000**
Dyspnea	0-100	8.4 (17.1)	11.3 (17.9)	.090	8.1 (16.7)	9.4 (18.8)	.326	.848	.311
Insomnia	0-100	22.0 (28.3)	26.5 (29.7)	.076	18.1 (24.6)	18.6 (24.4)	.788	.162	**.006**
Appetite loss	0-100	13.8 (23.1)	13.7 (24.0)	.990	6.8 (15.0)	7.6 (16.1)	.523	**.001**	**.005**
Constipation	0-100	8.2 (19.0)	15.2 (25.5)	**.001**	12.8 (21.8)	12.4 (21.3)	.761	**.030**	.253
Diarrhea	0-100	3.3 (11.3)	3.5 (10.9)	.862	3.3 (10.0)	4.8 (14.1)	**.**217	.970	.347
Financial difficulties	0-100	17.7 (29.0)	37.9 (34.3)	**.000**	10.3 (22.7)	8.7 (19.4)	.309	**.008**	**.000**
**Sense of Coherence**	13-91	67.2 (11.3)	63.1 (13.4)	**.000**	61.9 (14.0)	62.9 (13.4)	.171	**.000**	.870
**Spiritual Perspective Scale**	10-60	54.5 (4.9)	51.5 (6.5)	**.000**	49.0 (7.5)	49.2 (6.9)	.424	**.000**	**.001**
**Religious Coping (+)**	7-28	24.1 (4.2)	22.8 (4.3)	**.000**	22.8 (4.3)	22.9 (4.2)	.684	.**004**	.806
**Religious Coping (−)**	7-28	11.5 (4.7)	12.0 (4.9)	.135	12.3 (4.7)	12.0 (4.2)	.322	.072	.927

^1^BC: breast cancer; ^2^C: Control, ^3^Paired t-test; ^4^Independent Student’s t-test.

Bold values show statistical significant differences within and between the groups.

### Changes over time

Changes within the breast cancer group indicated that these women had poorer scores at T2 on the functional (physical, role, cognitive, and social functioning) and symptom scales (fatigue, nausea/vomiting, pain, constipation, and financial difficulties), in addition to the lower scores for the SOC, spirituality, and positive religious coping, compared with T1 (Table 3). However, they had better scores for global quality of life and emotional functioning at T2 than at T1. Within the control group, all scale scores remained stable between T1 and T2 (Table 3).

The differences between the two groups were significant for most outcome variables (lower functional and more symptom scales scores) in the breast cancer group compared with the controls at T2. But, the changes of SOC and religious coping scales scores were not significant and only the spirituality scale scores showed a significant decrease in the breast cancer group compared with the controls at T2 (Table 3).

When adjusting for the number of comorbidities, (GLM repeated measures), a significant interaction effect was found between time and group from T1 to T2 in the breast cancer group, compared with the controls, for some of the EORTC QLQ-C30 scale scores, and for the SOC, spirituality, and positive religious coping scales scores. In comparison with the controls, the breast cancer group scored lower on the physical, role, cognitive and social functioning scales scores, and reported more symptoms scale scores (fatigue, nausea/vomiting, pain, constipation, and financial difficulties) over time. Furthermore, the breast cancer group rated decreased scores in SOC, spirituality, and positive religious coping, but increased scores on global quality of life and emotional functioning over time from T1 to T2. Impaired areas of physical and role functioning, more fatigue and financial difficulties were clinically significant in the breast cancer group, compared with the controls over time (Table 4).

**Table 4:** **The results of the GLM repeated measures and clinical changes over time according to the adjusted mean differences, for EORTC QLQ-C30, SOC, SPS, and RCOPE (+) and RCOPE (−) scale scores in the breast cancer group (n = 162) compared to the controls (n = 210) from baseline (T1) to the 6-month follow-up (T2)**

**Variables**	**GLM results** **interaction effect** ^**a**^	**Statistical changes over time** **(p value)**	**Adjusted mean differences between groups over time** ^**b**^ **(Clinical changes)**
	Group * Time	**T1 → T2**	**T1 → T2**
**EORTCQLQ-C30:**			
Global quality of life	12.5	**.000**	+8.2
**Functional scales:**			
Physical	118.5	**.000**	**- 16.2**
Role	30.1	**.000**	**- 11.0**
Emotional	9.8	**.002**	+8.5
Cognitive	7.9	**.005**	- 6.0
Social	8.4	**.004**	- 7.0
**Symptom scales:**			
Fatigue	22.7	**.000**	**+12.0**
Nausea/vomiting	4.7	**.030**	+3.4
Pain	13.3	**.000**	+8.6
Dyspnea	0.6	.419	+1.7
Insomnia	1.7	.184	+4.0
Appetite loss	0.1	.707	- 1.0
Constipation	8.6	**.004**	+7.5
Diarrhea	0.4	.545	- 1.1
Financial difficulties	51.7	**.000**	**+21.7**
**Sense of Coherence**	17.3	**.000**	- 6.5
**Spiritual Perspective Scale**	31.0	**.000**	- 6.6
**Religious Coping (+)**	9.8	**.002**	- 6.5
**Religious Coping (−)**	3.5	.060	+4.3

In the last column plus and minus signs represent a higher or lower scoring on the respective variable.

Higher scoring (+) for symptom scales and RCOPE (−) scale shows impairment over time.

^**a**^GLM repeated measures tests adjusted for the number of the comorbidities.

^b^Differences in adjusted mean score in the breast cancer group (T2 - T1) minus differences in adjusted mean score in the controls (T2 - T1) show clinical changes

(Criterion: small 5–10, moderate 11–19, and large ≥ 20).

Bold values show significant statistical and clinical changes in the breast cancer group compared to the controls over time.

All scales were linearly transformed to 0–100.

### Predictors of HRQoL in the breast cancer group at T2

The results of the MLRs (Table 5) showed that the strongest predictors of the EORTC QLQ-C30 scales were the degree of SOC (p < 0.01) and baseline ratings of several dimensions of HRQoL (p < 0.05), 6 months after the pre-diagnosis phase of breast cancer. The spirituality and religious coping scores did not predict the scales of the EORTC QLQ-C30.

**Table 5:** **The results of the multiple linear regression analyses for HRQoL dimensions, summary of the significant predictors for the scales of the EORTC QLQ-C30 in the breast cancer group (n = 162) at the 6-month follow-up**

**Dependent variables**	$\widehat{\boldsymbol{\upbeta}}$	**p value**	**Dependent variables**	$\widehat{\boldsymbol{\upbeta}}$	**p value**
**Global quality of life**			**Pain**		
Education	0.21	**.003**	Disease stage	0.17	**.032**
SOC	0.50	**.000**	SOC	-0.26	**.003**
**R** ^**2**^ = 0.33			PA-baseline	0.20	**.012**
**Physical Functioning**			**R** ^**2**^ = 0.16		
Disease stage	-0.19	**.012**	**Dyspnea**		
SOC	0.31	**.000**	DY-baseline	0.26	**.001**
PF-baseline	0.27	**.000**	**R** ^**2**^ = 0.16		
**R** ^**2**^ = 0.26			**Insomnia**		
**Role Functioning**			SOC	-0.39	**.000**
Disease stage	-0.17	**.036**	SL-baseline	0.36	**.000**
SOC	0.33	.**000**	**R** ^**2**^ = 0.30		
**R** ^**2**^ = 0.19			**Appetite Loss**		
**Emotional Functioning**			Education	-0.27	**.001**
Surgery type	-0.22	**.001**	Disease stage	0.17	**.034**
SOC	0.49	**.000**	SOC	-0.27	**.001**
**R** ^**2**^ = 0.40			**R** ^**2**^ = 0.17		
**Cognitive Functioning**			**Constipation**		
Age	0.16	**.041**	CO-baseline	0.25	**.001**
SOC	0.26	.**002**	**R** ^**2**^ = 0.13		
RCOPE (−)	-0.17	**.034**	**Diarrhea**		
**R** ^**2**^ = 0.21			Disease stage	0.27	**.001**
**Social Functioning**			**R** ^**2**^ = 0.12		
SOC	0.33	**.000**	**Financial Difficulties**		
**R** ^**2**^ = 0.25			SOC	-0.26	**.002**
**Fatigue**			FI-baseline	0.28	**.000**
Disease stage	0.15	**.041**	**R** ^**2**^ = 0.24		
SOC	-0.36	**.000**			
FA-baseline	0.21	**.005**			
**R** ^**2**^ = 0.25					

CO: constipation; DY: dyspnea; FA: fatigue; FI: financial difficulties; PA: pain; PF: physical functioning; RCOPE (−): negative religious coping; SL: insomnia; SOC: sense of coherence.

Coding of the independent variables: age (continuous variable), education (lower education: high school and lower levels; higher education: college and university), disease stage (mild: 0 to II or severe: III and higher), surgery type (mastectomy or conservation surgery), and treatment modalities at the 6-month follow-up (ongoing treatment or treatment ended).

$\widehat{\upbeta}$: Standardized regression coefficient.

## Discussion

With the objective to determine why pattern of changes in HRQoL differ between studies during the illness trajectory for patients with breast cancer, we hypothesized that obtaining pre-diagnosis ratings could be one way of exploration. In that way all women are in a similar situation at baseline. In an ideal situation the recruitment should have been from a national mammography screening program, but this does not exist in Iran. Hence, the present study compared women in Iran, who were referred to the surgical wards for symptoms suggestive of breast cancer and diagnosed as having breast cancer later on, with a matched group of women who were visiting the Mammography Center, and were not diagnosed subsequently with breast cancer.

The baseline measurements differed between the groups. The women in the breast cancer group rated their global quality of life and emotional functioning worse, but they were less physically affected. These results could be explained by the women referred for surgery and final diagnosis being in a more threatening situation [69] than the controls, who visited Mammography Center by referral from a physician, either for screening or other reasons. On the other hand, fear of a cancer diagnosis and adverse psychological reactions have been reported in women who were referred for screening mammography programs as well [70,71]. A study found that women with early-stage breast cancer, in comparison to women among the general population reported poorer emotional, cognitive, and social functioning as well as more insomnia, appetite loss, and diarrhea at baseline, but less pain and financial difficulties [13]. In our study the degree of SOC, spirituality, and positive religious coping were rated higher in the breast cancer group than in the controls at baseline. This might depend on the threat of the disease influencing the women early, a noticeable finding in the care process. Women with breast cancer have earlier shown high spirituality and religious coping [72,73]. Hence, with regard to Iran as a predominantly religious-spiritual society [56], these differences are not surprising. There is a lack of normative data for SOC, spirituality, and religious coping scores in the Iranian population. However, in comparison with a healthy sample collected in the same region [62], the women with breast cancer had higher SOC, spirituality, and positive religious coping scores. In contrast, the controls were closer to the norm mean values [62].

Patients within the breast cancer group reported impaired functions and increased symptoms 6 months after the baseline measure, in contrast to improved perception of global quality of life and emotional functioning. A similar pattern has been reported in a prospective study without a control group, 3 months after initial treatment in a sample of Iranian women with breast cancer [65]. In parallel to our study, a Scandinavian study reported decreased physical, role, and social functions, and increased emotional functioning and fatigue among women with breast cancer. However, those women had a decreased global quality of life up to 25 weeks after chemotherapy, which was contrary to our findings [74]. Probably, most patients recover from their first emotional reactions during the first 6 months [14], although the impact of different adjuvant treatments and their side effects are likely to increase [18]. In our study, at the 6-month follow-up only a small proportion of the patients were in the treatment endpoint phase which was controlled for in the regression analyses. However, other prospective studies within the first 6 months showed different results. A prospective study with a 3-month follow-up after diagnosis showed no change in overall quality of life, physical functioning, fatigue, nausea/vomiting, and financial difficulties of patients with breast cancer in comparison to the general population, although role, emotional, cognitive, and social functioning deteriorated and pain decreased [13]. The findings of a 2-year follow-up study indicated that the greatest improvements appeared in almost all HRQoL dimensions during the first 6 months after surgery [22]. The diversity among study results may depend on several factors such as various treatment regimens and different side effects, absence of a control group, applying different instruments for assessing HRQoL, and the time of baseline and follow-ups in relation to diagnosis. Our study and the only other study using EORTC with a pre-diagnosis baseline (without a control group and also from Iran) showed similar results at baseline [65].

The most important impairment areas for women with breast cancer, compared with the controls over time were physical and role functioning, fatigue and financial difficulties. These areas have previously been distinguished as being areas of concern in some studies, both during and after treatment [15,65]. Therefore, it is important to plan for the impact of the disease trajectory and the effects of adjuvant treatments on the patients’ HRQoL and its dimensions [7]. Physical and role impairment as well as fatigue may continue for a long period postoperatively and threaten the patients’ independence [75]. Financial difficulties were highly prominent in our study, a finding that has been reported both in high-income and in low/middle-income countries [15,76]. Financial burden may result from several causes, including work absence, costs of treatment and traveling to and from the hospital [77]. It can be argued that despite of coverage of at least one kind of health insurance for more than about 90% of Iranian inhabitants, and governmental support of health costs, out-of-pocket expenditure on health remains as high as 55% for the Iranian population [78].

After adjustment for the number of the comorbidities, the degree of SOC, spirituality and positive religious coping showed a small decrease in the breast cancer group in comparison to the controls over time, but these changes were not clinically important. These small changes make it uncertain whether these variables are stable or change over time. There are probably minor or slow changes in the level of SOC after exposure to stressful situations, as Antonovsky explained [24]. Furthermore, these data present initial evidence that spirituality and religious coping may be sensitive to changes during the disease diagnosis and treatment phases, but this needs more consideration through longitudinal studies with different samples [38,79].

After controlling for demographic data and the treatment modalities at the 6 months follow-up, the degree of SOC and baseline ratings of several dimensions of HRQoL were the most important variables, predicting changes in the level of both some physical and mental dimensions of HRQoL 6 months after the pre-diagnosis period of breast cancer. It has been reported that the SOC seems to be stronger correlated to the mental dimension than the physical [33]. In our study the predictive role of the SOC for both types of dimensions of HRQoL is almost comparable. The importance of a pre-diagnosis rating of HRQoL dimensions in women with breast cancer becomes more obvious here. It has been recognized that baseline ratings of HRQoL can be predictive of health outcomes, such as survival rate, and response to the treatment in cancer patients over time [80,81]. In our study higher baseline ratings of physical functioning impairment and several symptoms were the most important predictors for the same HRQoL dimensions 6 months after the pre-diagnosis phase of breast cancer.

The value of SOC as an important predictor of HRQoL changes has been supported by the results of several studies [26,27,82]. Higher SOC as an inner resource may imply a stress buffering power, which contributes to better adaptive abilities in specific situations [24]. The power of spirituality and religious attitudes on the prediction of HRQoL has been shown in several studies in a Muslim context [55,83,84]. But in our study, the influence of the degree of SOC was more pronounced than spirituality and religious coping. This is in line with the theory of SOC, emphasizing the importance of an individual’s overall view of life on how to manage strain, rather than special coping strategies [24]. Probably cancer opens many demands in a patient’s life [7]. The role of the SOC can be regarded as the ability to find and utilize resources. Key components in the concept of SOC (comprehensibility, manageability and meaningfulness) are the resources that are available to supply energy and assist individuals to cope with the demands of stressful life events. Thus, the SOC as an inner resource is more clearly concentrated on factors promoting health rather than factors that refer to particular diseases [24]. Similar results regarding the SOC were found in an earlier study by the authors, examining SOC, spirituality and religious coping simultaneously as predictors of well-being within a healthy sample of the Iranian population [62].

Despite the potential effect of spirituality on HRQOL in different studies [85,86], we didn’t find any significant correlation in this study. A possible interpretation for why the spirituality was not a predictor in our study is that often the questionnaires measuring spirituality have a distinction between an existential (a feeling of meaning, peace, and connection to the self and others) and a religious dimensions (the belief and experience of connection with a higher power). The existential dimension appears to be more associated with HRQoL [86] and well-being than the religious dimension [47]. However, even if this shows that dimensions of spirituality are individual, it does not mean spirituality has considerable overlap with core HRQoL dimensions, because the correlations are not strong [87]. In our study, we chose the SPS in which the items are categorized to reflect spiritual behaviors and beliefs, but the SPS yields a single score and Reed [46,64] who developed the SPS did not discuss the subscales [63]. Therefore, our results underscore that the degree of SOC as an overall view of life and coping capacity, may be more important than spirituality and religion, as a general predictor for HRQoL changes. These findings suggest that testing the longitudinal role of the SOC as a mediator or moderator in the prediction of HRQoL in future research could contribute to the knowledge base for the SOC in the psychological adaptation of patients with breast cancer.

Baseline measurements at the pre-diagnosis phase together with a matched control group selection, is one of the strengths of this study, but there are several limitations. The groups did not differ after frequency matching regarding age, education, marital and working status, menopausal status, and existence of comorbidities. However, there were differences in the baseline HRQoL variables between the groups, which could be related to fear of the diagnosis, surgery, and cancer treatment in the breast cancer group and/ or a higher number of comorbidities reported by the controls. The difference in the number of comorbidities may be due to the fact that data were obtained from medical records in the breast cancer group, but by a self-report questionnaire in the control group. The reason being limited access to medical records of the control group, as they were not patients at the hospitals after mammography. It should be noted that subjective reports of comorbidities could bring greater variation of the results, in comparison with a validated record-based system [88]. This may also indicate that the women already in hospital care, because of symptoms suggestive of breast cancer, may not be fully comparable to the women who sought or were referred to mammography, as also reflected in the degree of SOC. Another potential bias was that data gathering was done in the hospitals at T1. But at T2, the questionnaires were posted by express mail, because some patients were living in other cities, and would not visit the hospitals in Tehran after initial treatment, as follow-up treatments were done in local clinics. The drop-out rate may have increased dramatically, if the patients had to visit the hospital once again just to fill out the questionnaires.

## Conclusions

During the first 6 months after pre-diagnosis, physical and role functioning, fatigue, and financial difficulties were prominent areas of impairment in HRQoL dimensions in a sample of breast cancer patients compared with a matched control group of women. In contrast, during the same time span, the women with breast cancer improved in overall quality of life and emotional functioning. The degree of sense of coherence and baseline ratings of several dimensions of HRQoL are important predictors for patient HRQoL changes at the 6-month follow up rather than spirituality and religious coping. The study corroborates Antonovsky’s suggestion regarding cross-cultural applicability of the sense of coherence concept in prediction of HRQoL changes. Our study results support the importance of collecting data concerning HRQoL and sense of coherence early at the pre-diagnosis period of breast cancer. Gathering sense of coherence data together with HRQoL data can assist in early detection of women who may be at greater risk for HRQoL impairments and have lower ability to adapt to the disease and treatment psychologically.

## Abbreviations

HRQoL: Health-Related Quality of Life
SOC: Sense of Coherence
SPS: Spirituality Perspective Scale
Brief RCOPE: Brief Religious Coping
EORTC: European Organization for Research and Treatment of Cancer
GLM: General Linear Model
MLR: Multiple Linear Regression
